# Platelet-rich plasma alone is unable to trigger contact osteogenesis on titanium implant surfaces

**DOI:** 10.1186/s40729-022-00427-1

**Published:** 2022-06-06

**Authors:** Ung-Gyu Kim, Jung-You Choi, Jun-Beom Lee, In-Sung Luke Yeo

**Affiliations:** 1 Department of Prosthodontics, School of Dentistry and Dental Research Institute, Seoul National University, 101 Daehak-ro, Jongro-gu, Seoul, 03080 Korea; 2 Dental Research Institute, Seoul National University, Seoul, Korea; 3 Department of Periodontology, Seoul National University School of Dentistry, Seoul, Korea

**Keywords:** Dental implants, Osseointegration, Bone morphogenetic proteins, Platelet-rich plasma

## Abstract

**Purpose:**

Osseointegration consists of bidirectional bone formation around modified implant surfaces by contact osteogenesis and distance osteogenesis. This study tested whether contact osteogenesis on the surface of a modified titanium (Ti) implant is stimulated by cytokines in the blood.

**Methods:**

In the first two types of experiments, sandblasted, large-grit, acid-etched Ti implants and turned Ti tubes were inserted into rabbit tibiae. To exclude the influence of distance osteogenesis, the tubes were inserted into the tibiae, and implants were placed inside the tubes. In a third type of experiment, the implants and tubes were inserted into the rabbit tibiae, and platelet-rich plasma (PRP) or recombinant human bone morphogenetic protein-2 (rhBMP-2) was applied topically. Four weeks after implantation, undecalcified specimens were prepared for histomorphometry. Bone-to-implant contact (BIC) and bone area per tissue (BA) were measured, and the data were analysed using one-way ANOVA at a significance level of 0.05.

**Results:**

When the response of bone to Ti tubes with implants was compared to that without implants (first experiment), little bone formation was found inside the tubes. The mean BIC of implant specimens inside the tubes was 21.41 ± 13.81% in a second experiment that evaluated bone responses to implants with or without Ti tubes. This mean BIC value was significantly lower than that in the implant-only group (without tubes) (47.32 ± 12.09%, *P* = 0.030). The third experiment showed that rhBMP-2 significantly increased contact osteogenesis on the implant surface, whereas PRP had no effect (mean BIC: 66.53 ± 14.06% vs. 16.34 ± 15.98%, *P* = 0.004).

**Conclusions:**

Platelet-rich plasma alone is unable to trigger contact osteogenesis on the modified titanium implant surface.

## Background

The insertion of a dental implant, typically composed of titanium (Ti), is the primary option for replacement of a missing tooth. The structural and functional connection between living bone and metal implants, known as osseointegration, was first described by Per-ingvar Brånemark in the 1960s [1]; however, the nature of the contact between bone and the surface of a Ti implant remains unknown. Some authors have described an affinity between bone and Ti, while others have interpreted osseointegration as an immunologic response to the metal surface [2, 3]. When a dental implant is inserted into the jawbone, bone formation and direct contact of the bone with the implant surface are accomplished by two mechanisms: contact osteogenesis and distance osteogenesis [4]. In contact osteogenesis, bone formation begins on the implant surface and extends towards the bone, whereas distance osteogenesis involves the formation of new bone on the surface of existing bone and its subsequent progression towards the implant surface.

In the field of implant dentistry, topographical or chemical modifications of Ti implant surfaces are well known to induce contact osteogenesis and to accelerate osseointegration [5–10]. However, a previous in vivo study showed that contact osteogenesis was largely eliminated when sandblasted, large-grit, acid-etched (SLA) implants and hydrophilic SLA implants were inserted into rabbit tibiae within a Ti tube, blocking the effects of other factors, such as bone morphogenetic proteins, from existing bone, despite a continued blood supply [11]. Interestingly, in a previous in vivo study using fluorochrome labelling and rabbit femurs, distance osteogenesis was unaffected by chemical and topographical modifications of the surface properties of dental implants, whereas there were significant differences in bone-to-implant contact (BIC) between the modified surfaces, suggesting that the quality and efficiency of osseointegration depend largely on contact osteogenesis [12]. Contact osteogenesis on dental implant surfaces is achieved by modifications of implant surfaces, blood supply, and factors derived from existing bone, whereas distance osteogenesis requires only a blood supply containing factors from existing bone [5–12]. In particular, it has been thought that factors from existing bone might be necessary to trigger contact osteogenesis. Bone morphogenetic protein-2, one of the cytokines stored in and released from existing bone, is known to promote bone formation when the bone is wounded or fractured [13, 14]. In dental implants, bone morphogenetic protein-2 is also known to stimulate bone formation around dental implants [15].

The aim of our current study was to test whether contact osteogenesis on a modified Ti implant surface could be achieved by blood-borne factors alone. To this end, we used a rabbit tibial implant model, which has the advantages of easy access to the surgical site, fast metabolism of the experimental animal, and a short lifespan of the experimental animal [16]. Platelet-rich plasma (PRP) was used to examine the effect of blood-borne factors. PRP is known to contain many growth factors and cytokines that stimulate bone formation and soft tissue healing [17–19]. Fontana et al. described the effect of PRP on the peri-implant bone response: the use of platelet-rich plasma showed a significant increase in peri-implant bone volume [20]. The hypothesis underlying this study was that blood-borne factors alone can promote contact osteogenesis on modified Ti implant surfaces.

## Methods

### Preparation of specimens

Thirty-nine implants with a diameter of 3.0 mm and a length of 12.0 mm were prepared by Deep Implant System, Inc. (Seongnam, Korea) using a computer numerical control system (CINCOM L20; Citizen Machinery Co., Ltd., Kitasaku-gun, Japan). The implants were screw-shaped and made of grade 4, commercially pure Ti, and the surfaces were modified by sandblasting with alumina particles and etching with hydrochloric acid. A sandblasting pressure of 400 kPa and a hydrochloric acid concentration of approximately 11.5 M were used to produce an SLA surface. To separate the implants from bone-derived substances in the in vivo experiments, 24 Ti tubes were constructed using the CINCOM L20 system. The tubes were made of commercially pure grade 4 Ti and had the following dimensions: outer diameter, 4.0 mm; inner diameter, 3.6 mm; thickness, 0.2 mm; and length, 6.0 mm. Unlike the implants, the tubes had no surface modifications.

### Surface characteristics of the implants and Ti tubes

Six implants and six Ti tubes underwent surface analysis using field emission scanning electron microscopy (FE-SEM; S-4700; Hitachi, Tokyo, Japan) to generate overall surface images and X-ray photoelectron spectroscopy (Sigma Probe; Thermo Scientific, Waltham, MA, USA) to analyse surface composition. Three implants and three tubes were used to analyse surface topology. Confocal laser scanning microscopy (LSM 5-Pascal; Carl Zeiss AG, Oberkochen, Germany) was performed to measure the surface roughness. The surface roughness parameters Sq (root mean square height of the surface), Sa (arithmetical mean height of the surface), and Sdr (developed area ratio) were chosen based on previous studies [21–23]. Additionally, Ra, which is the one-dimensional version of Sa, was measured for each specimen. The measurement of every surface parameter was performed on three spots of each specimen; upper, middle and lower surface areas were arbitrarily selected. The measured area of each spot was 100 μm × 100 μm, and the mean of the values measured at the three spots was the surface parameter value of each specimen.

### In vivo surgery

#### Surgical procedures

The animal experiments performed in this study were conducted under the guidelines of the Institutional Animal Care and Use Committees (IACUC, #201805001) of CRONEX Co., Ltd. (Hwaseong, South Korea) and were performed in accordance with the Animal Research: Reporting of In Vivo Experiments 2.0 (ARRIVE 2.0) guidelines for in vivo animal experiments [24]. Eight male New Zealand white rabbits (*Oryctolagus cuniculus*), aged 1–2 years and weighing 2.6–3.0 kg, were used.

**Fig. 1:**
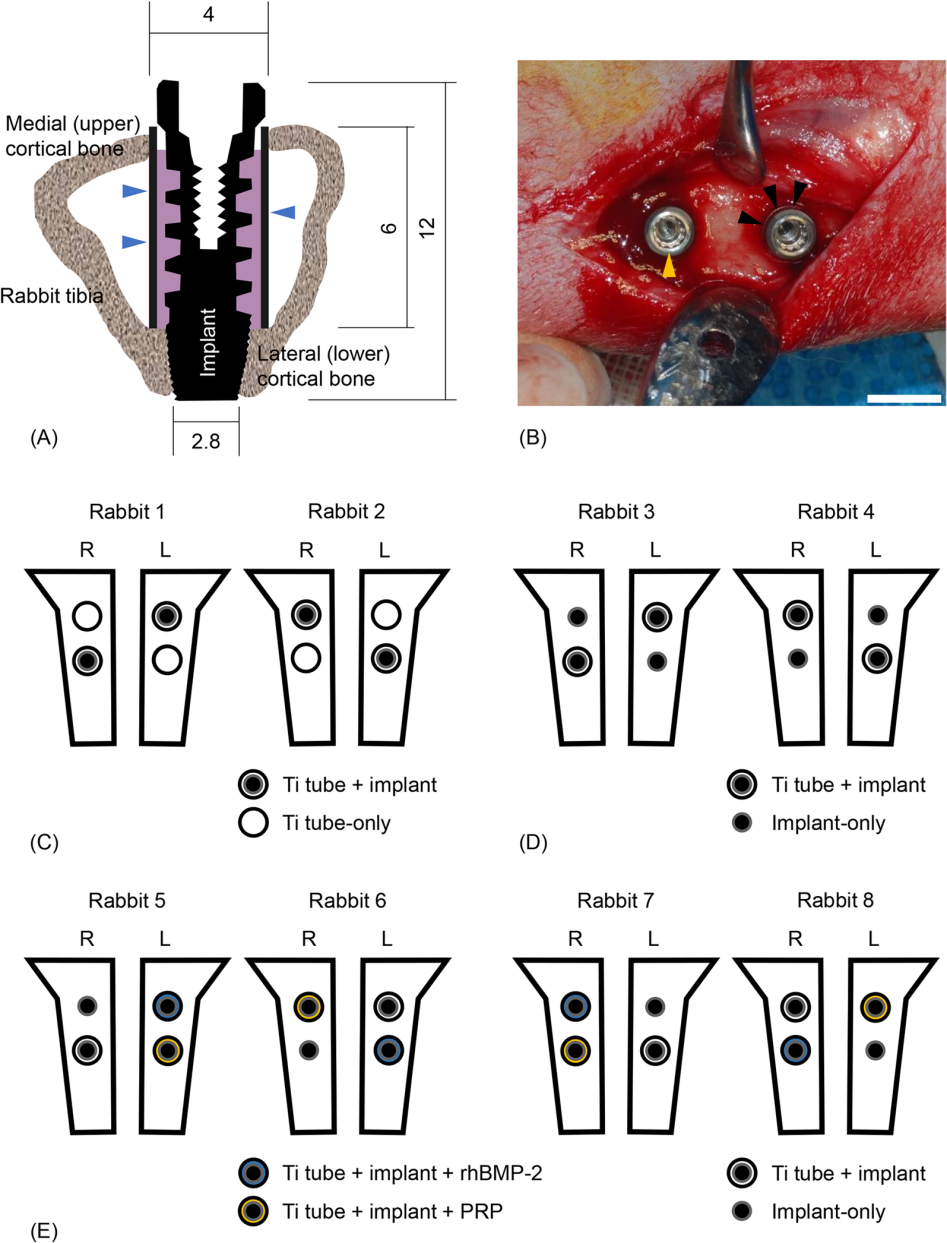
**A** Schematic drawing of an implant and tube (blue arrowheads) inserted into a rabbit tibia. The tibia was drilled bicortically, the tube was inserted into the upper cortical bone, and the implant was inserted through the tube into the lower cortical bone. The gap (pink area) between the implant and the tube allowed blood flow from the lower cortical bone. Numbers indicate dimensions in millimetres. **B** Two implants were inserted into each tibia. An implant (yellow arrowhead) is shown on the left without a Ti tube, while on the right, a Ti tube (black arrowheads) is shown around the implant. Scale bar = 5 mm. **C** Schematic diagram comparing the response of bone to implants in Ti tubes with that to Ti tubes alone. The experimental groups were arranged in a 2 × 2 Latin square. **D** Schematic diagram of the comparison of the response of bone to implants with or without Ti tubes. The groups were arranged in a 2 × 2 Latin square. **E** Evaluation of the effects of blood-borne substances on bone formation. A split-plot design was used for this experiment. RhBMP-2 was used in the Ti tube + implant + rhBMP-2 group to represent cytokines derived from existing bone. PRP from each rabbit was applied to the Ti tube + implant + PRP group to test the effects of blood-borne factors on bone formation. *R* right tibia, *L* left tibia, *Ti* titanium, *rhBMP-2* recombinant human bone morphogenetic protein-2, *PRP* platelet-rich plasma

All rabbits were anaesthetized via intravenous injection of tiletamine/zolazepam (15 mg/kg; Zoletil® 50; Virbac Korea Co., Ltd., Seoul, Korea) and xylazine (5 mg/kg; Rompun; Bayer Korea, Ltd., Seoul, Korea). The skin over the proximal tibia was shaved and washed with betadine, after which a cephalosporin antibiotic (50 mg/kg cefazolin; Yuhan Co., Seoul, Korea) was administered intramuscularly. Subsequently, 0.9 mL of 2% lidocaine HCl (Huons Co., Ltd., Seongnam, Korea) with 1:100,000 epinephrine was injected locally into the two surgical sites of each animal. The skin was then incised, and the tibia was exposed by muscle dissection and periosteal elevation. Drills and profuse sterile saline irrigation were used to prepare the implant sites on the flat medial tibial surface. Drilling was performed bicortically, with a final diameter of 4 mm in the upper (medial) cortical bone (used to compare the experimental and control groups) and 2.8 mm in the lower (lateral) cortical bone (the anchorage site for implant placement) (Fig. 1A and B). After implant insertion, the muscle and fascia were sutured with resorbable 4–0 Vicryl sutures, and the outer dermis was closed with nylon sutures. Each rabbit was housed separately after surgery, and the antibiotic cephalosporin was administered intramuscularly for 3 days.

#### Bone response to Ti tubes with or without SLA implants

In the first experiment, the response of bone-containing Ti tubes with inserted implants was compared with that of bone-containing tubes alone. For Ti tubes with implants, a hole was prepared to insert the tube into the upper cortex of the tibia. The tubes were inserted into the prepared holes (4 mm in diameter) in the upper cortex of the tibiae; subsequently, the implants were inserted and anchored to the lower cortex (2.8 mm diameter) (Fig. 1A and B). In the Ti tube-only group, the tubes were inserted into the holes, but no implant was placed. Two rabbits were used for this experiment, and each tibia contained two surgical sites. A two-by-two Latin square arrangement was used to ensure complete randomization of the arrangement of the experimental groups, as shown in Fig. 1C.

#### Bone response to SLA implants with or without Ti tubes

In a second experiment, the response of bone-containing implants within Ti tubes was compared with that of bone-containing implants alone. The bones with implants ± Ti tubes were prepared as described above. In the implant-only group, the implants (3 mm in major diameter) were inserted through the upper holes (4 mm in diameter) and anchored to the lower cortex; however, no tubes were inserted. Two rabbits were used, and each tibia contained two surgical sites. The arrangement of the experimental groups was completely randomized using the two-by-two Latin square design (Fig. 1D).

#### Evaluation of the effects of substances from blood and bone on bone formation

In the third experiment, to evaluate the effects of substances from blood and bone on bone formation, we included an implant-only group (positive control), an implant + tube group (negative control), an implant + tube + rhBMP-2 group, and an implant + tube + PRP group, where in the last two groups, rhBMP-2 or PRP was applied to both the implant and the wounded bone after implantation (Fig. 1E). PRP was selected as a source of blood-borne factors because PRP is derived from the blood of each individual and contains high concentrations of various osteogenic cytokines [25–28]. RhBMP-2 was selected to represent one of the existing bone-derived factors, despite not being from the experimental animals in this study, because bone morphogenetic proteins, BMP-2 in particular, are highly osteoinductive growth factors released from old bone matrix, and a previous immunohistochemical analysis of bone samples surrounding Ti implants revealed high levels of BMP-2 [11, 13, 29–32].

Four rabbits were used, and as in the other in vivo experiments, each tibia contained two surgical sites. This experiment included two control (implant-only and Ti tube + implant) and two experimental (Ti tube + implant + rhBMP-2 and Ti tube + implant + PRP) groups. The Ti tube + implant group served as a negative control, and the implant-only group served as a positive control. The first experimental group had an implant within a Ti tube, to which rhBMP-2 was applied (CowellMedi, Busan, South Korea). In this group, a portion of 0.1 mL rhBMP-2 solution (0.25 mg/mL) was applied to the implant surface before implantation, and the remainder was injected between the Ti tube and the implant after implantation. The second experimental group had an implant within a Ti tube, to which PRP was applied. PRP was prepared using the protocol described by Pazzini et al. [33]. Briefly, rabbit blood was collected in a tube containing sodium citrate as an anticoagulant and centrifuged at a relative centrifugal field (RCF) of 40 × *g* for 10 min. After discarding red blood cells, the sample was centrifuged at 626 × *g* for an additional 10 min. Subsequently, PRP was collected and applied to the implant and bone defects after implantation. The methods used for insertion of the implants and tubes were as described for the first and second experiments. The groups were arranged in the rabbit tibiae according to a split-plot design (Fig. 1E).

### Histomorphometry

After a 4-week healing period, all of the experimental animals were anaesthetized and killed via an intravenous overdose of potassium chloride. The tibiae were exposed, and a section of bone containing the inserts was surgically removed en bloc with a bone collar. The bone blocks containing the inserts were immediately fixed in 10% neutral formaldehyde. Specimens were prepared for histomorphometry using the method of Choi et al. and Donath and Breuner [11, 34]. Briefly, the bone samples with inserts were embedded in light-curing resin (Technovit 7200 VLC, Kulzer, Wehrheim, Germany), and undecalcified specimens were sliced and ground to a thickness of less than approximately 50 μm using the EXAKT system (EXAKT Apparatebau, Norderstedt, Germany) and stained with haematoxylin and eosin (H&E). The H&E staining used in this study was processed routinely for general histological evaluation [35]. The stained specimens were analysed using a light microscope (Olympus BX, Olympus, Tokyo, Japan). Image analysis software (ImageJ, U.S. National Institutes of Health, Bethesda, Maryland, USA) was used to calculate the BIC and BA ratios of the best three consecutive threads from the light microscope images for each implant [36].

### Statistical analysis

Statistical analyses were performed using R software (version 3.6.1; R Foundation for Statistical Computing, Vienna, Austria). Data were analysed for normality with the Shapiro–Wilk test. *P* > 0.05 indicated statistical normality. Data are expressed as the mean and standard deviation (SD). For the BIC and BA data, one-way analysis of variance (ANOVA) was performed to identify significant differences among the groups. When ANOVA indicated a significant difference, pairwise comparisons were conducted using Duncan’s post hoc test. *P* < 0.05 indicated statistical significance.

## Results

### Surface characteristics of Ti implants and tubes

**Fig. 2:**
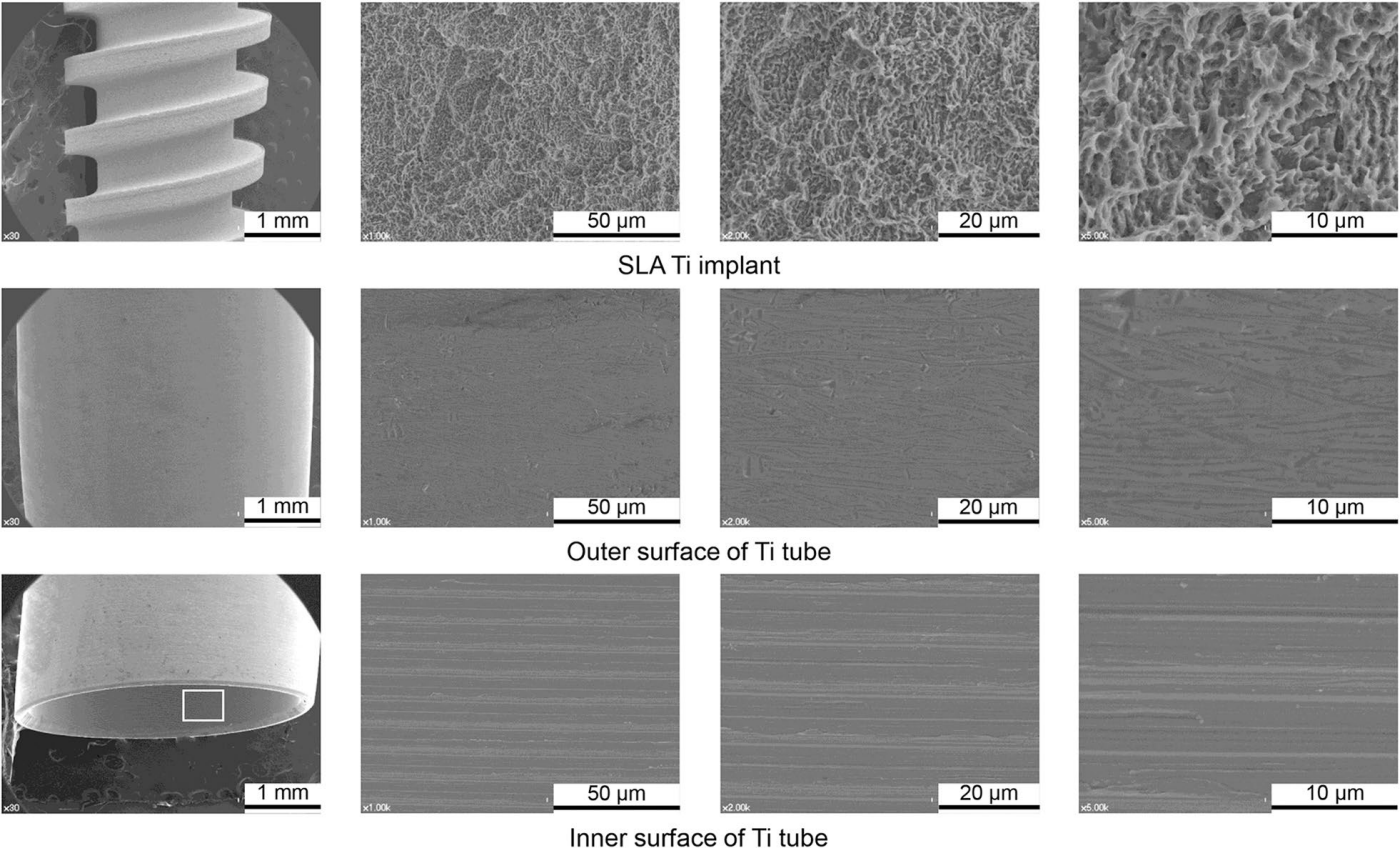
Surface characteristics of SLA Ti implants and turned Ti tubes. Representative scanning electron microscopy images of the SLA Ti implants and the turned Ti tubes. The white rectangle indicates the inner surface of the Ti tube

**Fig. 3:**
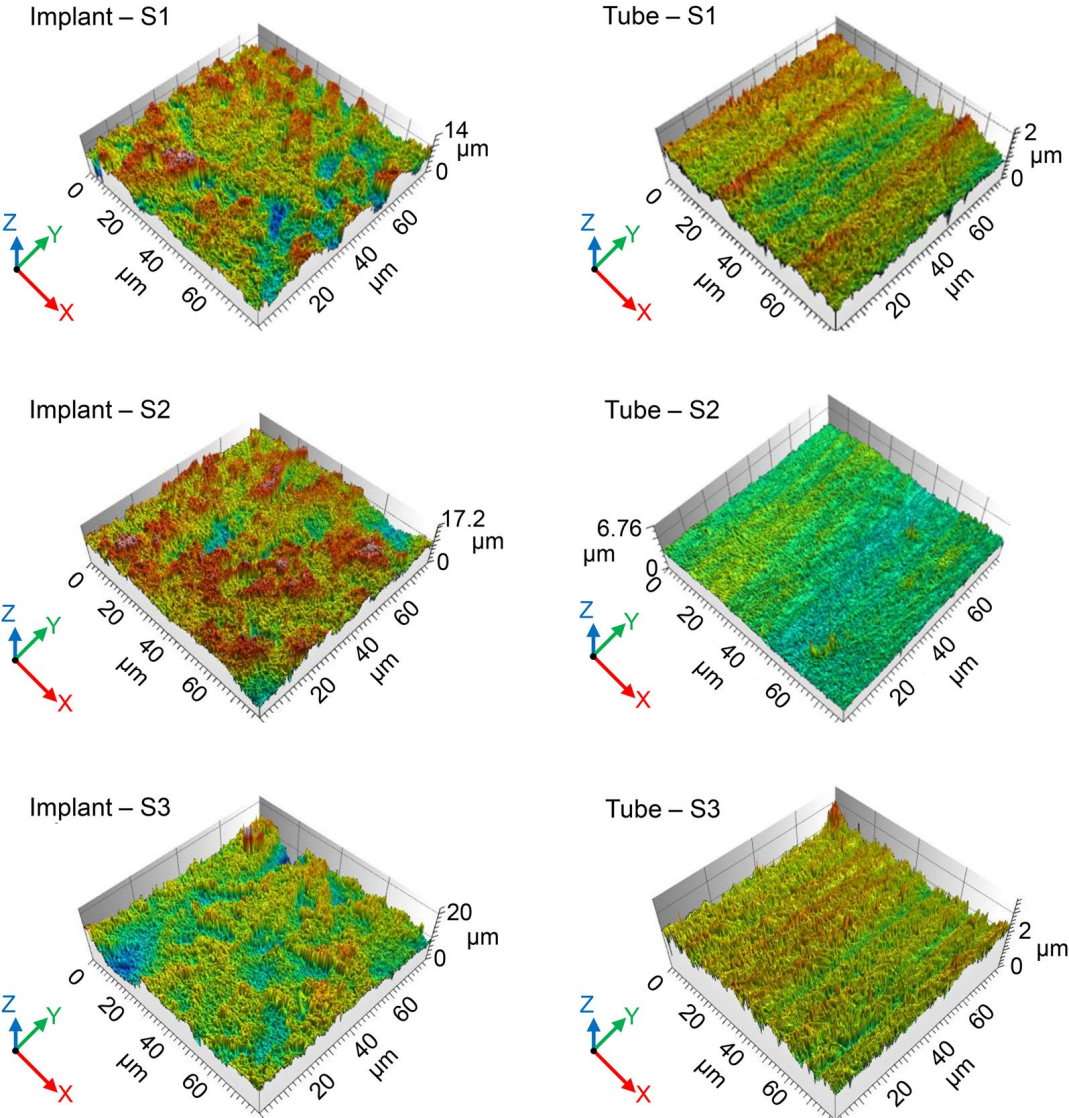
Confocal laser scanning microscopy of the surface of implant and tube specimens. Notice that the SLA implant specimens (left) have rougher surfaces than the turned or unmodified tube specimens (right), particularly in the Z-axis. *S* specimen

The surface characteristics of the Ti implants and tubes were analysed because contact osteogenesis occurs only on a microtopographically modified Ti surface, not on the smoother surface of turned Ti [4]. After all the screw-shaped Ti implants were prepared, their surfaces were modified by SLA for contact osteogenesis. The Ti tubes were constructed to physically block the flow of substances from existing bone in vivo, but their surfaces were not modified by SLA. FE-SEM analyses of the SLA-modified surfaces of the Ti implants revealed honeycomb-shaped irregularities (Fig. 2, top row). The inner and outer surfaces of the Ti tubes displayed a grooved pattern, which is a characteristic feature of the computer-controlled milling process used to manufacture the tubes (Fig. 2, middle and bottom rows). Analysis of confocal laser scanning microscopy images also showed that the SLA-modified surfaces of the implants were significantly rougher than those of the unmodified Ti tubes (Table 1, Fig. 3). Subsequent X-ray photoelectron spectroscopy of the turned surfaces of the tubes and SLA-modified surfaces of the implants showed that they consisted mainly of Ti and oxygen, with mean levels (atomic %) of 24.30 ± 0.35% and 75.70 ± 0.35%, respectively, for the implants and 16.36 ± 1.30% and 83.64 ± 1.30%, respectively, for the tubes.

**Table 1:** The surface roughness parameters of the SLA Ti implants and turned Ti tubes

Implant	Sq (μm)	Sa (μm)	Ra (μm)	Sdr (%)
S1	1.45	1.12	0.80	175
S2	1.30	1.01	1.08	147
S3	1.39	1.09	0.98	157
Mean	1.38*	1.07*	0.95*	160*
SD	0.08	0.06	0.14	14.2

Tube	Sq (μm)	Sa (μm)	Ra (μm)	Sdr (%)
S1	0.093	0.071	0.061	2.85
S2	0.136	0.096	0.080	3.90
S3	0.145	0.109	0.087	8.54
Mean	0.125	0.091	0.076	5.10
SD	0.028	0.019	0.013	3.03

*S* specimen, *SD* standard deviation

*Significantly higher (*P* < 0.05)

### Bone response to Ti tubes and to SLA Ti implants inside the tubes

**Fig. 4:**
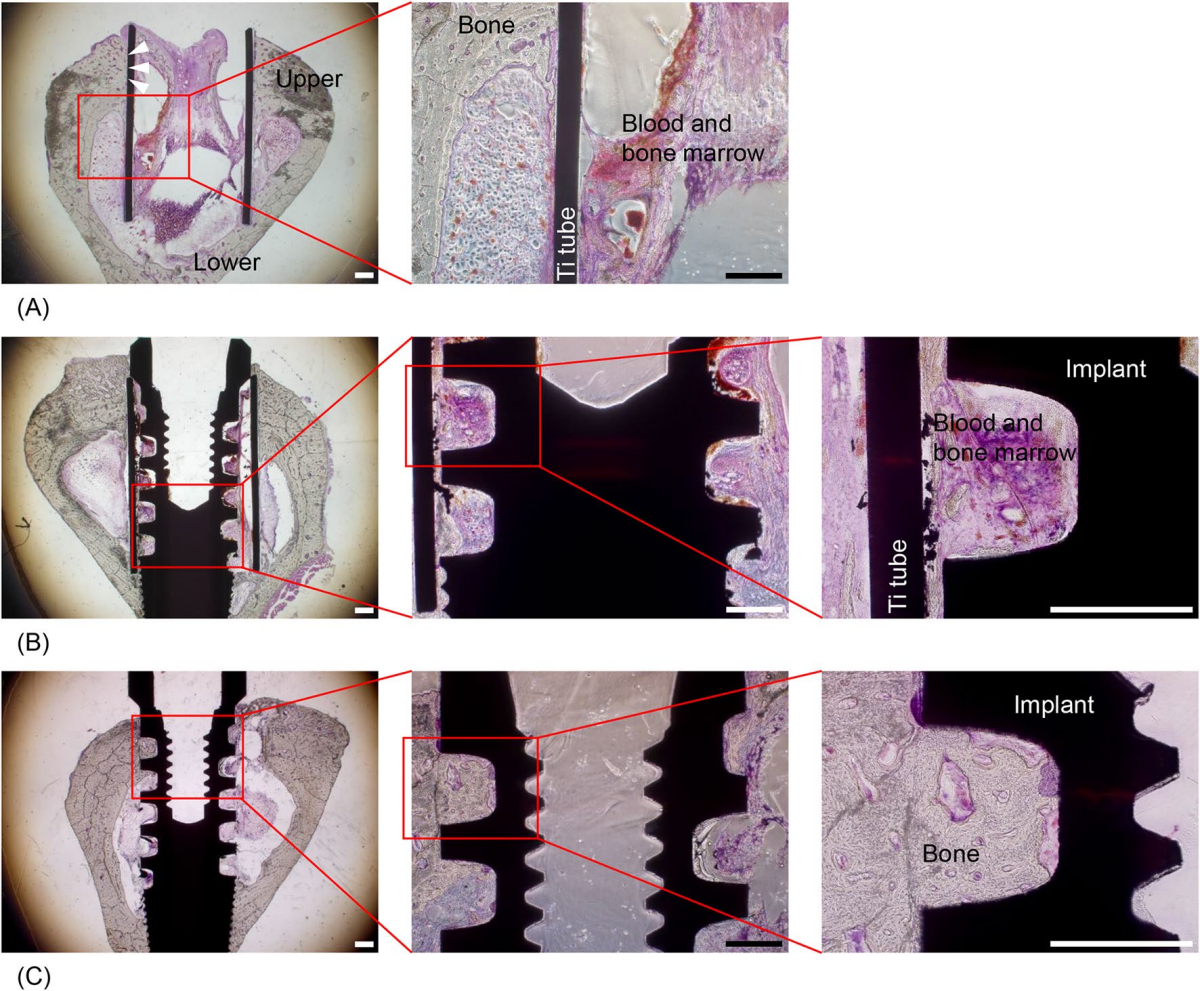
Representative histological images (haematoxylin and eosin staining) of bone formation around the SLA Ti implants and the turned Ti tubes. **A** Ti tube-only condition. Normal bone (grey stain) healing on the turned outer surface is shown (white arrowheads). Inside the tube, however, little bone formation was observed, even 4 weeks after implant insertion into the rabbit tibia. **B** Ti tube + implant condition. Little or no bone formation was observed between the Ti tube and the Ti implant. Most of the area between the tube and the implant was filled with blood and bone marrow (purple stain). **C** With no Ti tube (implant-only condition), normal bone formation was observed around the SLA Ti implants. Scale bars = 500 μm

For the first experiment where the response of bone-containing Ti tubes into which implants were inserted was compared to that in the absence of implants, the BIC ratio in the Ti tube-only group was not measured, as there was no implant in this group, but the bone formation pattern was monitored and qualitatively evaluated. Little bone formation was observed in the group that only had Ti tubes, inside of which mainly blood and bone marrow were observed, and outside of which there was normal contact of cortical bone with the turned Ti surface. Bone formation started in the lower region of the rabbit tibia, but very little bone formation occurred in the upper cortical area (Fig. 4A). When SLA Ti implants were inserted into the Ti tubes, almost no bone formation occurred between the SLA surface of the Ti implants and the turned surface of the Ti tubes (Fig. 4B).

For the second experiment, which compared the response of bone that contained implants with that in the absence of Ti tubes, the histological findings for specimens containing implants within Ti tubes were similar to those observed in the experiment described above (Fig. 4B). Normal bone formation occurred in the implant-only group, filling the space between the implant threads with new bone (Fig. 4C). To quantitatively evaluate contact osteogenesis on the SLA Ti implant surface, histomorphometry was carried out to calculate the mean BIC and BA ratios. A Shapiro–Wilk test was performed to check the normality of the BIC and BA data and did not show evidence of non-normality (*P* > 0.05). The mean BIC ratio in the implant-only group was 47.32 ± 12.09%, which was significantly higher than that in the implant + tube group, where the mean BIC ratio was 21.41 ± 13.81% (*P* = 0.030). When BIC was measured for the Ti tube + implant under conditions identical to those in the first experiment above, the calculated mean value was similar, 19.13 ± 6.45%. The mean BA ratio in the implant-only group was 35.62 ± 5.40%, which showed no significant difference from the implant + tube group, where the mean BA ratio was 12.16 ± 8.17% (*P* = 0.077).

### Inability of blood-borne substances to trigger contact osteogenesis on SLA Ti implant surfaces

**Fig. 5:**
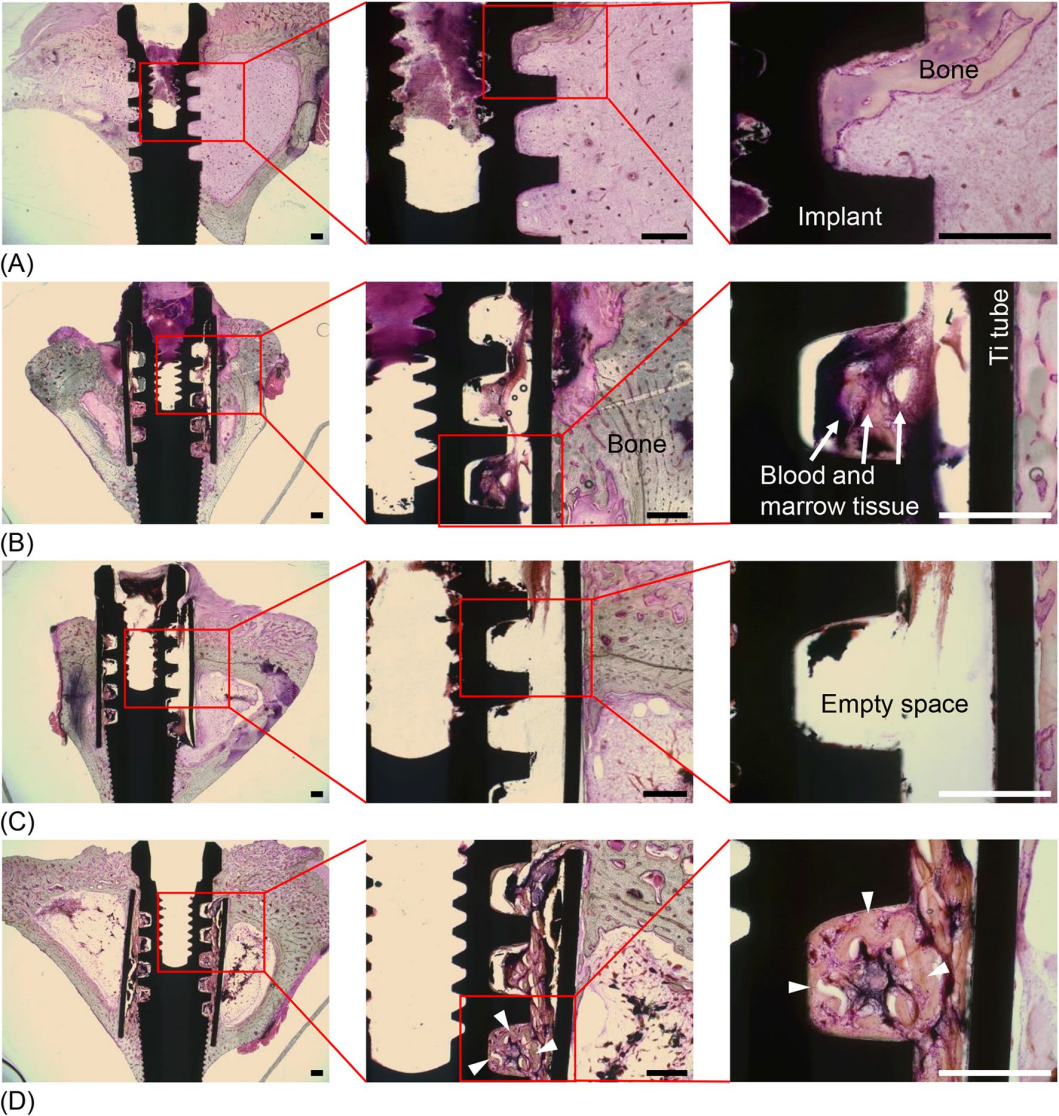
Representative histological images (haematoxylin and eosin staining) of bone formation in the experiment to evaluate the influence of substances from blood and bone. **A** In the implant-only group, normal bone formation was observed around the SLA Ti implants. **B** In the Ti tube + implant group, there were many areas between the implant threads with no bone formation. **C** In the Ti tube + implant + PRP group, no bone formation was observed between many of the implant threads despite the PRP injection. **D** In the Ti tube + implant + rhBMP-2 group, bone formation (white arrowheads) was clearly observed. Scale bars = 500 μm

Histologically, bone formation in both the implant-only and Ti tube + implant groups in these experiments was similar to that in the experiments described above (Fig. 5A and B). General bone contact on the SLA Ti implant surface was observed in the implant-only group, while minimal contact osteogenesis was observed on the SLA Ti implant surface within the Ti tube. Interestingly, PRP had no significant effect on bone formation between the Ti tubes and the SLA Ti implants in the Ti tube + implant + PRP group (Fig. 5C). Despite PRP from each experimental animal being injected into the surgical site, contact osteogenesis was rare, similar to the results in the Ti tube + implant group. Notably, bone formation was stimulated when rhBMP-2 was applied inside the Ti tubes, where contact osteogenesis on the SLA Ti implants was clearly observed (Fig. 5D).

A Shapiro–Wilk test was performed to check the normality of BIC and BA data and did not show evidence of non-normality (*P* > 0.05). Histomorphometrically, the mean (± SD) values of the measured BIC ratios were as follows: implant-only, 53.33 ± 6.09%; Ti tube + implant, 33.22 ± 9.83%; Ti tube + implant + PRP, 16.35 ± 15.98%; and Ti tube + implant + rhBMP-2, 66.53 ± 14.06% (Table 2). The differences among the four groups were significant (*P* = 0.004, one-way ANOVA), and Duncan’s post hoc test revealed that the BIC ratio in the Ti tube + implant + rhBMP-2 group was significantly higher than those in the Ti tube + implant and Ti tube + implant + PRP groups. The mean (± SD) values of the measured BA ratios were as follows: implant-only, 17.60 ± 10.82%; Ti tube + implant, 28.13 ± 5.87%; Ti tube + implant + PRP, 15.86 ± 11.33%; and Ti tube + implant + rhBMP-2, 35.92 ± 5.35% (Table 3). The differences among the four groups showed no statistical significance (*P* = 0.068, one-way ANOVA).

**Table 2:** Bone-to-implant contact (BIC) ratios in the third experiment (*n* = 3)

Treatment	Implant only	Implant + tube	Implant + tube + PRP	Implant + tube + rhBMP-2
	54.87	24.74	0	51.37
BIC (%)	46.62	44.00	17.11	79.12
	58.50	30.91	31.94	69.09
Mean	53.33	33.22	16.35	66.53*
SD	6.09	9.83	15.98	14.06

*SD* standard deviation

*Significantly higher (*P* < 0.05)

**Table 3:** Bone area per tissue (BA) ratios in the third experiment (*n* = 3)

Treatment	Implant only	Implant + tube	Implant + tube + PRP	Implant + tube + rhBMP-2
	21.22	31.42	18.90	35.45
BIC (%)	26.15	22.27	3.32	41.50
	5.43	30.70	25.36	30.83
Mean	17.60	28.13	15.86	35.93
SD	10.82	5.09	11.33	5.35

*SD* standard deviation

## Discussion

This study aimed to test whether contact osteogenesis on a modified Ti implant surface can be promoted by blood-borne factors alone. Bone regeneration around an implant comprises two processes: contact osteogenesis and distance osteogenesis. To examine whether contact osteogenesis is dependent on substances from bone or blood, we used a Ti tube to physically isolate implants inserted into rabbit tibiae that blocked the influx of substances from existing bone. We concluded that blood-borne cytokines alone did not promote contact osteogenesis on the SLA Ti implant surface. The Ti tubes used in our experiments not only blocked the effects of existing bone (distance osteogenesis), but also limited the blood supply to the implant by allowing blood flow only from the bottom of the cortical bone [11]. In the experiment comparing the response of bone to the insertion of Ti tubes containing SLA Ti implants with that of Ti tubes alone, little bone formation was observed in the Ti tube-only group, although some did occur in the lower region of the tube, likely due to the release of substances from existing bone in that region. In the experiment comparing the responses of bone to the insertion of SLA Ti implants with or without Ti tubes, bone regeneration around implants within a Ti tube was much less than that around implants alone. The results of these two experiments indicate that physically blocking the effects of existing bone using a Ti tube reduces bone regeneration around the implant. Similar results were reported in a previous study [11].

To determine whether blood-derived factors could cause contact osteogenesis, we examined the effect of applying PRP to implants inside tubes on the BIC ratio. In addition, we hypothesized that a factor derived from existing bone, BMP-2, is necessary for contact osteogenesis based on the results of a previous study [11]. To test this hypothesis, we examined the effect of applying rhBMP-2 to implants inside tubes on the BIC ratio. New osteogenesis in the Ti tube + implant + rhBMP-2 group was similar to that in the positive control (implant-only) group and was substantially higher than that in the negative control (Ti tube + implant) group. PRP did not enhance bone formation around implants in Ti tubes. These results suggest that BMP-2 released from existing bone might initiate contact osteogenesis on the implant surface. Consistent with such a role for BMP-2, previous studies have shown that it plays an important role in bone development and can stimulate the production of new bone [37, 38]. In a recent study, Alhussaini examined the effects of platelet-rich fibrin (PRF), which is more concentrated in healing factors than PRP, and BMP-2 on the implant stability quotient [39]. At the time of implant insertion, there were no significant differences among the implant-only (control), implant + PRF, and implant + rhBMP-2 groups. However, at 6 and 12 weeks after surgery, implant stability was significantly higher in the rhBMP-2 group than in the control or PRF groups. Similarly, in our experiments, bone regeneration around the SLA Ti implant inside the turned Ti tube increased significantly when rhBMP-2, but not PRP, was applied, indicating that blood-borne cytokines alone are unable to trigger contact osteogenesis on the modified Ti implant surface. These results suggest that a factor derived from existing bone, probably BMP-2, was necessary for bone formation around the SLA Ti implant surface.

We added PRP to the bone–implant interface. PRP contains a number of growth factors (GFs), such as vascular endothelial GF, platelet-derived GF, transforming GF-β, epidermal GF, insulin-like GF-1, and hepatocyte GF, that stimulate osteogenesis [17–19], and consequently, PRP is used in various treatments to promote bone regeneration. Marx et al. described an enhancing effect of PRP on the density of bone grafts of mandibular continuity defects; specifically, PRP significantly increased trabecular bone density [40]. Another study concluded that PRP may improve the outcome of grafting in sinus floor elevation and alveolar ridge preservation, as well as reducing postoperative pain and swelling [19]. Despite these findings, in our experiments, the BIC ratio was clearly higher in the Ti tube + implant + rhBMP-2 group than in the Ti tube + implant + PRP group. In addition, PRP did not promote bone regeneration around the implant in our experiments using Ti tubes to block the effects of existing bone, contrary to reports in earlier studies when factors released from wounded bone were mixed with PRP or PRF [19, 26, 41]. Although blood-borne factors may be unable to initiate bone formation in the absence of factors from bone, they may help to promote osteogenesis after initiation of the healing process.

The rabbit tibia model used in this study has advantages for study of the osseointegration process after implant placement. The rabbit has Haversian systems similar to those of humans and has the advantages of easy access to the surgical site and fast bone metabolism and a short lifespan of the experimental animal, allowing analysis of the osseointegration period within a short time [16]. In this study, we did not apply a load to implants placed in the rabbit tibia, and the healing period of 4 weeks after the placement of implants was suitable to simulate the early osseointegration stage of the healing period in humans [42].

Subsequent X-ray photoelectron spectroscopy of the Ti tube and SLA-modified surface of implants showed significantly lower Ti and higher oxygen contents of the tubes (both *P* values < 0.05). This may have been due to the oxide layer on the tube surface. However, considering the surface characteristics reported in previous studies, all the turned Ti tubes and SLA Ti implants were acceptable for in vivo implantation [3, 43, 44].

To block the effect of existing bone, a Ti tube was inserted into the tibia. We drilled the hole in the upper cortex to insert the Ti tube, and due to the irregularity of the rabbit tibia, the Ti tube might contact the lower cortex of the rabbit tibia. A 2D histologic slide was used to measure the distance between the Ti tube and the lower cortex of the tibia. The average distance between the Ti tube and the lower cortex was 0.71 ± 0.90 mm.

We used Koch’s postulates, proposed by Robert Koch in 1890, to evaluate the possible causal relationship between BMP-2 and contact osteogenesis. Koch’s postulates include the following four criteria, which were originally designed to establish a causal relationship between a microorganism and a disease: (1) the microorganism must be present in every case of the disease; (2) the microorganism must be isolated from the diseased organism and grown in pure culture; (3) a pure culture of the microorganism should cause the disease when inoculated into a healthy susceptible organism; and (4) the microorganism must be re-isolated from the inoculated, diseased experimental host and identified as being identical to the original specific causative agent [45]. A previous study using immunohistochemical analyses demonstrated that BMP-2 is located along the interface between newly formed bone and an implant [11]. Assuming that BMP-2 could be isolated from the bone–implant interface, Koch’s first and second postulates are considered to be proven. Our finding that the application of rhBMP-2 to the interfacial area increased the BIC ratio, indicating the promotion of new bone growth, satisfies the third postulate. Regarding the final postulate, BMP-2 was not re-isolated from new bone in this study. This issue of re-isolation should be investigated in future research. Additionally, other factors originating from existing bone should be evaluated; BMP-2 was selected in this study as a candidate to trigger contact osteogenesis based on several previous studies.

## Conclusions

Within the limitations of this in vivo study, we concluded that platelet-rich plasma alone is unable to trigger contact osteogenesis on a modified Ti implant surface. Our findings from these in vivo experiments suggest that existing bone-derived factors may be needed for bone formation around an implant and that bone-derived BMP-2 can initiate contact osteogenesis. The results of this study provide insight into the mechanism of contact osteogenesis on a modified Ti surface to form a hard tissue system. Further research is needed to identify biological and molecular mechanisms involved in osteogenesis and to find ways to improve the clinical prognosis of dental implants.

## Data Availability

All data generated or analysed during this study are included in this published article.

## Abbreviations

ANOVA: Analysis of variance
BIC: Bone-to-implant contact
FE-SEM: Field emission scanning electron microscopy
GF: Growth factor
PRP: Platelet-rich plasma
RCF: Relative centrifugal field
RhBMP-2: Recombinant human bone morphogenetic protein-2
SD: Standard deviation
SLA: Sandblasted, large-grit, acid-etched
Ti: Titanium
